# Ficoll-based isolation biases the profiling of immune cells and leukemic blasts in AML samples

**DOI:** 10.3389/fimmu.2026.1920697

**Published:** 2026-08-25

**Authors:** Bianca E Silva, Alison Daubry, Charline Faville, Adrien De Voeght, Jacques Foguenne, Mégane Jassin, Oswin Kwan, Leslie Correia Da Cruz, Gabriele Carriglio, Sébastien Charles, Alexandra Veloso, Frédéric Baron, Jo Caers, André Gothot, Grégory Ehx

**Affiliations:** 1 GIGA Institute, Laboratory of Hematology, University of Liège, Liège, Belgium; 2 Department of Hematology, University Hospital Center of Liège, Liège, Belgium; 3 Department of Laboratory Hematology, University Hospital Center of Liège, Liège, Belgium; 4 Walloon Excellence in Life Sciences and Biotechnology (WELBIO) Department, WEL Research Institute, Wavre, Belgium

**Keywords:** AML, Ficoll, granulocytes, hemolysis, leukemic stem cells, T cells

## Abstract

**Introduction:**

An increasing number of studies focus on anti-tumor immune responses. In acute myeloid leukemia (AML), blasts and immune cells, such as T cells, are frequently studied together in the same leukocyte sample. While hemolysis is the clinical standard for leukocyte isolation because it preserves the native leukocyte composition, Ficoll-based density gradient centrifugation is widely used in research and biobanking.

**Methods:**

Using hemolysis as a reference, we assessed whether Ficoll processing introduces biases into AML and immune cell profiling through analytical methods frequently applied in AML research.

**Results:**

Flow cytometry analyses showed that Ficoll systematically alters the composition of AML samples by enriching lymphocytes and AML blasts while depleting granulocytes. In RNA sequencing analysis, Ficoll isolation altered the expression of 1,136 genes, notably leading to an overestimation of the expression of leukemic stem cell gene sets. Immunogenomic deconvolution highlighted that Ficoll leads to an overestimation of CD8^+^ T-cell and monocyte abundances. Mutation calling from RNA-seq data revealed substantial discrepancies between methods, including failure to detect a clinically relevant DNMT3A R882 mutation in a Ficoll-processed sample. While Ficoll isolation had minimal impact on *ex vivo* AML blast expansion or chemotherapy response, it appeared to affect AML engraftment in NSG mice, possibly through the enrichment of T cells mediating graft-versus-host disease.

**Discussion:**

Together, these findings show that Ficoll isolation introduces bias in the cellular and molecular characterization of AML samples while having only a moderate impact on functional assays. We provide recommendations on which method to use depending on the investigator's objectives.

## Introduction

Acute myeloid leukemia (AML) encompasses multiple subtypes characterized by the clonal proliferation of myeloid precursors (blasts) exhibiting various degrees of differentiation (1). Diagnosis and classification into AML subtypes and risk groups rely on the identification of multiple immunophenotypic and genetic markers, such as CD34 and CD117, mutations in FLT3-ITD, NPM1, and DNMT3A, as well as KMT2A (MLL) rearrangements (2). Given the high heterogeneity of AML and the complexity of the diagnostic process, strict guidelines established by the European LeukemiaNet (ELN), International Consensus Classification, and the World Health Organization must be carefully followed to ensure diagnostic accuracy and consistency across clinical centers (1–3).

Immunophenotyping by flow cytometry is a pivotal technique for characterizing AML and monitoring measurable residual disease (MRD) (4). While analytical procedures on untouched blood or bone marrow samples are under development, standard analyses require the removal of red blood cells to enrich for leukocytes and enable antibody staining. The currently recommended method for this enrichment is hemolysis [recommendation B4 of the ELN2021 (5) and others (6, 7)], which involves selectively lysing red blood cells using a hypotonic solution while sparing leukocytes, which tolerate osmotic stress more efficiently. This technique therefore preserves the entire leukocyte population without altering the relative abundance of subpopulations, making it the gold standard for routine clinical flow cytometry. Density-gradient centrifugation represents the main alternative to hemolysis for leukocyte isolation. In this approach, blood is layered over a polysaccharide-rich solution (most commonly Ficoll-Paque); upon centrifugation, red blood cells and granulocytes sediment, while peripheral blood mononuclear cells (PBMCs) remain at the plasma–Ficoll interface and are collected. While hemolysis is widely used in clinical diagnostics for AML, Ficoll separation is more commonly employed in biobanking (8, 9), large-scale multi-omics studies [e.g., Leucegene (10–13) and BEAT-AML (14)], and fundamental research (15–17).

The discrepancy in leukocyte isolation methods between clinical practice and experimental research prompted us to investigate the potential bias introduced by Ficoll-based isolation on the analytical approaches typically used to characterize AML samples in research, including flow cytometry, RNA sequencing (RNA-seq), *in vitro* functional assays, and engraftment in humanized mice. We hypothesize that Ficoll isolation may alter the proportions of non-leukemic immune cells within total leukocyte fractions isolated from AML patients. This effect is expected for granulocytes, but it may also affect smaller immune cell populations that could become artificially enriched as a consequence of granulocyte depletion. In the context of studies examining the abundance of immune cells [through flow cytometry or RNA-seq-based immunogenomic profiling (18–20)] in such samples, the use of Ficoll may therefore introduce a significant bias. Altered sample composition could also affect AML blast expansion dynamics in immunodeficient mice or in *ex vivo* assays. In addition to altering immune cell populations (which could indirectly affect the proliferative capacity of AML cells), Ficoll may alter the composition of the leukemic blast population or alter its overall abundance. Importantly, although the AML development in immunodeficient mice xenografted with human leukemic cells has traditionally been attributed to leukemic stem cells (LSCs) (16), leukemia-initiating activity has been observed outside the LSC fraction (21–24). Therefore, to maximize the representativeness of the leukemia reconstructed in xenografted animals, it may be preferable to transplant non-fractionated leukemic samples.

In this study, we observed that Ficoll isolation leads to an overestimation of leukemic (stem) cells and lymphocyte abundance while leading to an underestimation of granulocyte abundance in flow cytometry and RNA-seq-based immunogenomic analyses. Although this effect was found in all samples analyzed, its magnitude varied substantially from sample to sample. Ficoll isolation also had other impacts, such as inaccurate mutation calling, possible effects on AML engraftment capacity *in vivo*, and overestimation of AML proliferation capacity *in vitro*. To our knowledge, this is the first study to assess the amplitude of the distortion introduced by Ficoll isolation on the characterization of immune and leukemic cells in AML samples. Based on our observations, we generally recommend the use of hemolysis for studies in which the cellular composition of the sample is examined, while Ficoll may be preferable for studies focused on SSC^low^ blasts. We also provide considerations for selecting the most appropriate isolation method according to the intended downstream analysis.

## Methods

### Patient samples

Peripheral blood samples (~50 mL) from AML patients were obtained after written informed consent. Characteristics of the patients and samples are listed in **Table 1**.

**Table 1:** Characteristics of primary AML patient samples.

ID	Sex	Age	WHO 2022	ELN Risk	Disease status at sampling	Mutations	Blood WBC (10^3^/µl)	% Blasts
P41	F	80	AML, MDS-related	Unfavorable	Relapse	SF3B1, RUNX1, ASXL1	48.71	24
P42	F	63	AML, MDS-related	Unfavorable	Relapse	NRAS, SRSF2, RUNX1	50.47	20.5
P44	M	63	AML, MDS-related	Unfavorable	Diagnosis	DNMT3A, U2AF1, ASXL1, TET2	6.25	1.8
P45	M	56	AML with NPM1 mutation	Intermediate	Relapse	FLT3-ITD, NPM1	1.54	9.5
P46	F	64	AML, MDS-related	Unfavorable	Diagnosis	FLT3 Asp835Val, ASXL1, RUNX1, TET2	3.49	6
P47	F	41	AML, MDS-related	Unfavorable	Diagnosis	SAMD	1.93	1.8

White blood cell counts, blast percentages, and mutation data were obtained by our clinical department. ELN, European LeukemiaNet; MDS, Myelodysplastic Syndrome; WBC, white blood cells; WHO, World Health Organization.

### Leukocyte isolation

Equal volumes (~25 mL) of each peripheral blood sample were processed either for red blood cell lysis (hemolysis) or Ficoll-Paque gradient centrifugation. Hemolysis was conducted by incubating one volume of blood with four volumes of lysis buffer (25) (ammonium chloride (NH_4_Cl) 154.8 mM, EDTA 96.7 µM, potassium bicarbonate (KHCO_3_) 9.99 mM, diluted in ddH_2_O, pH 7.1, sterilized by autoclaving) for 15 min at room temperature under constant agitation. Leukocytes were then collected by centrifugation (500 ×g for 10 min) and washed in PBS. Ficoll-Paque gradient centrifugation was performed by layering blood samples (pre-diluted 2X in PBS) on 15 mL of Ficoll-Paque PLUS (cat: GE17-1440-02; Cytiva) and centrifuging for 20 min at 500×g without brake. PBMCs were collected by pipetting the mononuclear cell layer at the plasma–Ficoll interface and were washed with PBS. Isolated cells were counted with a Yumizen H500 hematology analyzer (Horiba).

### Immunophenotyping by flow cytometry

Cells were washed in PBS with 3% fetal bovine serum (FBS; cat: S-FBS-AU-015; Serana) before processing for flow cytometry staining. Cells were incubated with Fc receptor blocking solution (Human TruStain FcX, cat: 422302; Biolegend) for 10 minutes at room temperature before overnight incubation with surface antibodies at 4 °C, as recommended in (26). The following antibodies specific for human antigens were used (all purchased from Becton-Dickinson (BD) unless indicated otherwise): CD45-BV510 (clone HI30, cat: 563204); CD3-V450 (clone UCHT1, cat: 561812); CD19-APC (clone HIB19, cat: 17-0199-42; eBioscience); CD33-PE (clone HIM3-4, cat: 12-0339-42; eBioscience); CD15-FITC (clone W6D3, cat: 2215020; Sony Biotechnology); CD56-PE/Cyanine7 (clone HCD56, cat: 2191590; Sony Biotechnology); CD34-PerCP-Cy5.5 (clone 8G12, cat: 347222); CD117-APC-R700 (clone YB5.B8, cat: 565195). Cells were washed with PBS before analysis on a LSRFortessa flow cytometer (BD). Results were analyzed with FlowJo software v10 (Tree Star Inc.).

For mouse experiments, blood was collected by tail vein bleeding, lysed with RBC Lysis Buffer (cat: 00-4300-54; eBioscience), and washed in PBS with 3% FBS before staining. In addition to the above antibodies, the following antibodies were used: anti-human CD4-BV786 (clone SK3, cat: 563877); anti-human CD8-APC-Cy7 (clone SK1, cat: 348813); anti-mouse CD45-APC (clone 30-F11, cat: 559864). Cells were washed with PBS and incubated for 5 mins with 7-amino-actinomycin D (7-AAD, cat: 00-6993-50; eBioscience) before analysis on a cytoFLEX flow cytometer (Beckman Coulter (BC)).

Clinical routine analysis of immature myeloid populations included the use of the following antibodies specific for human antigens: CD34-FITC (clone 8G12, cat: 345801), CD13-PE (clone L138, cat: 347406), CD7-PerCPCy5.5 (clone M-T701, cat: 561602), CD33-PE/Cyanine7 (clone P67.6, cat: 333952), CD56-APC (clone NCAM16.2, cat: 341027), CD117- APC-A750 (clone 104D2D1, cat: B92450; BC), HLA-DR-Pacific Blue (clone Immu-357, cat: B36291; BC), and CD45-V500-C (clone 2D1, cat: 655873). Additional antigens were evaluated, such as anti-terminal deoxynucleotidyl transferase-FITC (HT1 + HT4 + HT8 + HT9, cat: IM3524; BC) and myeloperoxidase-PE (MPO; clone CLB-MPO-1, cat: B36288; BC), as well as CD300e-APC (clone UP-H2, cat: 656158), CD64-APCH7 (clone 10.1, cat: 561190), and CD36-V450 (clone CB38, cat: 561535) for determining monocytic lineage (2). The Boolean gating strategy of AML blasts for clinical analyses is illustrated in **Supplementary Figure 1**. This approach is based on the recommendations of the ELN for the immunophenotypic diagnosis of AML (5, 27). Blast cells are defined by a CD45^dim^/SSC^dim^ phenotype together with the expression of at least one immaturity marker (CD34 and/or CD117) and one or more myeloid lineage markers (CD117, CD13, CD33, and/or MPO). Exclusion of mature contaminating populations is achieved through the combined assessment of monocytic and lymphoid markers. The monocytic compartment is characterized by the expression of CD64, CD36, CD14, and CD300e. CD14 and CD300e are specific markers of terminal monocytic maturation, whereas CD64 and CD36 allow discrimination of the different stages of monocytic differentiation. Finally, T lymphocytes and NK cells are identified by the expression of CD7 and CD56, respectively.

### Mouse experiments

NOD-scid IL-2Rγ^null^ (NSG) mice (The Jackson Laboratory), aged 8–11 weeks, were administered an intravenous injection of 1×10^6^ leukocytes (isolated either by Ficoll or hemolysis) 24 h after 2.5 Gy total body irradiation. Graft-versus-Host Disease (GVHD) severity was assessed by a scoring system that incorporates 4 clinical parameters: weight loss, posture (hunching), mobility, and anemia, as previously reported (28). Each parameter received a score of 0 (minimum) to 2 (maximum). Mice were monitored daily and scored for GVHD thrice weekly. Mice with a score of 6/8, weight loss >20%, or displaying signs of distress were sacrificed in agreement with the recommendation of the ethics committee. Mice were euthanized with an intraperitoneal injection of 1520mg/kg of pentobarbital and 8mg/kg of lidocaine chloride (i.e. 200µl of pentobarbital 200mg/ml (Dolethal™, Vetoquinol, Niel, Belgium) mixed with 5% of lidocaine chloride 20mg/ml (Xylocaine ^®^, Aspen Pharma Trading Limited, Dublin, Ireland), for an average mouse weight of 25g), followed by cervical dislocation 15 minutes after the injection and only in the absence of breathing signs. The method for euthanasia was recommended by the Institutional Animal Care and Use Ethics Committee of the University of Liège, Belgium. Final scores for dead animals reaching the ethical limit score were kept in the data set for the remaining time points (last value carried forward).

### *Ex vivo* culture of primary AML cells

Following isolation by Ficoll or hemolysis, primary AML cells were seeded at a density of 1×10^6^ cells/mL in an initial volume of 1 mL per well in 24-well plates in the presence of murine bone marrow stromal cells (OP9 cells; cat: CRL-2749; American Type Culture Collection) as described in (18). Briefly, the OP9 feeder cells were pre-irradiated at 30 Gy and seeded at 80–90% confluence on 0.01% poly-D-lysine–coated plates (cat: A3890401; Gibco). Cells were transferred to a freshly irradiated OP9 feeder layer every 2 weeks or once the density exceeded 1.5×10^6^ cells/mL. Cultures were maintained in Iscove’s Modified Dulbecco’s Medium (cat: 12440053; Gibco) supplemented with 20% FBS, 1% penicillin-streptomycin (cat: 15140122; Gibco), and 50 µM of 2-mercaptoethanol (cat: 31350010; Gibco) at 37 °C in a humidified incubator with 5% CO_2_. A cytokine cocktail, consisting of SCF (50 ng/mL, cat: 300-07), G-CSF (20 ng/mL, cat: 300-23), GM-CSF (20 ng/mL, cat: 300-03), FLT3-L (50 ng/mL, cat: 300-19), IL-3 (20 ng/mL, cat: 200-03), and IL-6 (20 ng/mL, cat: 200-06), was added twice weekly (all cytokines from Peprotech).

Cell proliferation and viability were monitored thrice week by 7-AAD exclusion on a cytoFLEX flow cytometer. Before each measurement, the wells were carefully flushed to recover as many blasts as possible without detaching the OP9 stromal layer. Cultures were reseeded to 5×10^5^ cells/mL once this density was exceeded. Each subculture was documented to calculate the cumulative cell count at the end of the experiment, using the formula: cumulative cell count = cell count on the day × cumulative number of previous subcultures performed. If the cell viability dropped below 60%, the cells were centrifuged at 500×g for 5 minutes and resuspended in fresh medium supplemented with the cytokine cocktail.

### Drug preparation

Cytarabine (AraC; cat: HY-13605; MedChemExpress) and cytokines were reconstituted in PBS to the desired stock concentration. Sterile solutions were aliquoted and stored at -80 °C for up to six months.

### Analysis of senescence by flow cytometry

Cells were seeded at a density of 5×10^5^ cells/mL in 24-well plates and cultured in the presence of the cytokine cocktail but in the absence of the OP9 feeder layer. Immediately after seeding, the cells were treated daily with 1.5 µM AraC for three consecutive days. Staining with 5-Dodecanoylaminofluorescein Di-β-D-Galactopyranoside (C_12_-FDG; cat: HY-126839; MedChemExpress) was performed 24 h post-treatment with AraC. Briefly, the cells were washed by centrifugation (500×g, 5 min), and the pellet was resuspended in culture medium containing 100 nM bafilomycin A1 (cat: HY-100558; MedChemExpress), followed by incubation for 1 h at 37 °C and 5% CO_2_. Subsequently, the cells were incubated for 2 h with 33 µM C_12_-FDG or DMSO (cat: 67-68-5; Sigma-Aldrich) for unstained control conditions. After two washing steps, 7-AAD was added immediately before flow cytometry acquisition to assess C_12_-FDG fluorescence (FITC channel) exclusively in viable cells.

### RNA sequencing

After isolation, one million leukocytes were cryopreserved at -80 °C in TriPure Isolation Reagent (cat: 11667165001; Roche). Total RNA was isolated with the RNeasy Mini Kit (cat: 74106; Qiagen) according to the manufacturer’s instructions, and RNA quality and concentration were assessed using the 5200 Fragment Analyzer (Agilent Technologies). A total of 1 µg of RNA (RQN score >8) was processed using the NEBNext Ultra II Directional Poly(A) Library Prep Kit. Library profiles were verified on the Qiaxel Advanced (QIAgen) instrument using a DNA screening cartridge. The final dual-indexed libraries were quantified by qPCR using KAPA SYBR FAST (cat: KK4600; Roche) with Illumina standard and pooled equimolarly.

Sequencing was performed on the NovaSeq 6000 (Illumina) sequencer on an S4 flow cell, generating paired-end 150-cycle reads with a sequencing depth of 10 million fragments sequenced per sample. Raw sequencing data were demultiplexed using sample-specific indexes, filtered for quality, and converted into FASTQ files for downstream analysis using the bcl2fastq software. Sequencing data quality were assessed using the FastQC tool for individual reports per sample, and with MultiQC to generate a report comprising all samples.

### Bioinformatic analyses

Transcript expression quantifications were performed with kallisto v0.51.0 (29) in stranded mode. Kallisto’s transcript-level count estimates were converted into gene-level counts using the R package tximport v1.14.2. EdgeR v3.28.1 was used to normalize counts using the TMM algorithm and output counts per million (CPM) values. Differential gene expression analysis was conducted in R3.6.1 as reported previously (30). In brief, raw read counts were converted to CPM, normalized relative to library size, and lowly expressed genes were filtered by retaining genes with CPM >0 in at least two samples using edgeR (31) and limma (32) v3.42.2. Subsequently, voom transformations and linear modeling using limma’s lmfit were performed. Moderated t-statistics were then computed with eBayes. To perform a paired differential gene expression analysis, a custom contrast matrix was built with the model.matrix function to compare each Ficoll sample to its Lysis equivalent per patient. The contrast matrix was then used as input to the contrasts.fit function of limma. Differential expression was defined using an adjusted p-value <0.05 and an absolute log_2_ fold-change (FC) ≥1.

Gene set enrichment analysis (GSEA) was performed with fgsea package v1.12.0 in R (33). A pre-ranked gene list was generated by ranking expressed genes obtained from the paired limma–voom analysis on the moderated t-statistics. HALLMARK gene sets were obtained from the MSigDB database, and blood transcriptional modules (BTM) gene sets were obtained from (34).

Immune cell population deconvolutions were performed with CIBERSORTx, the default LM22 matrix and 100 permutations. Mutation analyses were performed by aligning raw RNA-seq reads on the GRCh38.113 genome with STAR v2.7.11a (35) running with default parameters to generate bam files. Single-base mutations with a minimum alternate count setting of 5 were identified using freeBayes v1.3.8 (36) and mutations were annotated with SnpEff v5.3a (37) and the clinVar database (downloaded on 31-05-2025) (38). Transcriptomic analyses performed on the BEAT-AML cohort (14) were conducted on publicly available data (https://biodev.github.io/BeatAML2/). Raw counts were converted to CPM values with EdgeR as indicated above and used as input of CIBERSORTx.

For immunogenomics analyses, CIBERSORTx deconvolution outputs were aggregated to approximate the cell populations defined by flow-cytometric gating. CD8^+^ T cells were defined as SSC^low^CD3^+^CD8^+^, whereas CD4^+^ T cells (including CD4 memory, CD4 memory activated, and CD4 naive subsets) were defined as SSC^low^CD3^+^CD4^+^. B-cell fractions (naive B cells, memory B cells, and plasma cells) corresponded to SSC^low^CD19^+^ cells, while resting and activated NK-cell fractions corresponded to SSC^low^CD56^+^ cells. Monocytes were defined as SSC^int^CD14^+^ cells, and granulocytes (including neutrophils and eosinophils) were defined as SSC^high^CD15^+^ cells. Samples were subsequently ranked according to the abundance of each population (using flow-cytometry–derived proportions among CD45^+^ cells for Lysis samples, or aggregated CIBERSORTx values for Ficoll and Lysis samples), and rankings were compared between the two methods for each isolation approach.

### Statistics

Unless specified in the figure legends, paired t-tests were used to compare two conditions, and correlations were assessed with the Pearson correlation coefficient. Boxplots display the median with 25^th^ and 75^th^ percentiles and whiskers extending to the 10^th^ and 90^th^ percentiles. Bar plots show the mean ± standard deviation (SD), when indicated. Plots and statistical tests were mainly performed with GraphPad Prism v10.6. For all statistical tests, ^∗∗∗∗^ refers to p < 0.0001, ^∗∗∗^ refers to p < 0.001, ^∗∗^ refers to p < 0.01 and ^∗^ refers to p < 0.05.

## Results

### Ficoll gradient isolation distorts leukocyte composition toward blasts and lymphocytes

To compare the impact of Ficoll isolation on AML characterization, six peripheral blood samples were freshly collected from random AML patients. Patient characteristics are summarized in **Table 1**. Equal parts of each sample were processed using either hemolysis (referred to as the “Lysis” condition hereafter) or Ficoll-based isolation (“Ficoll” condition hereafter), and the resulting samples were analyzed by flow cytometry.

**Figure 1:**
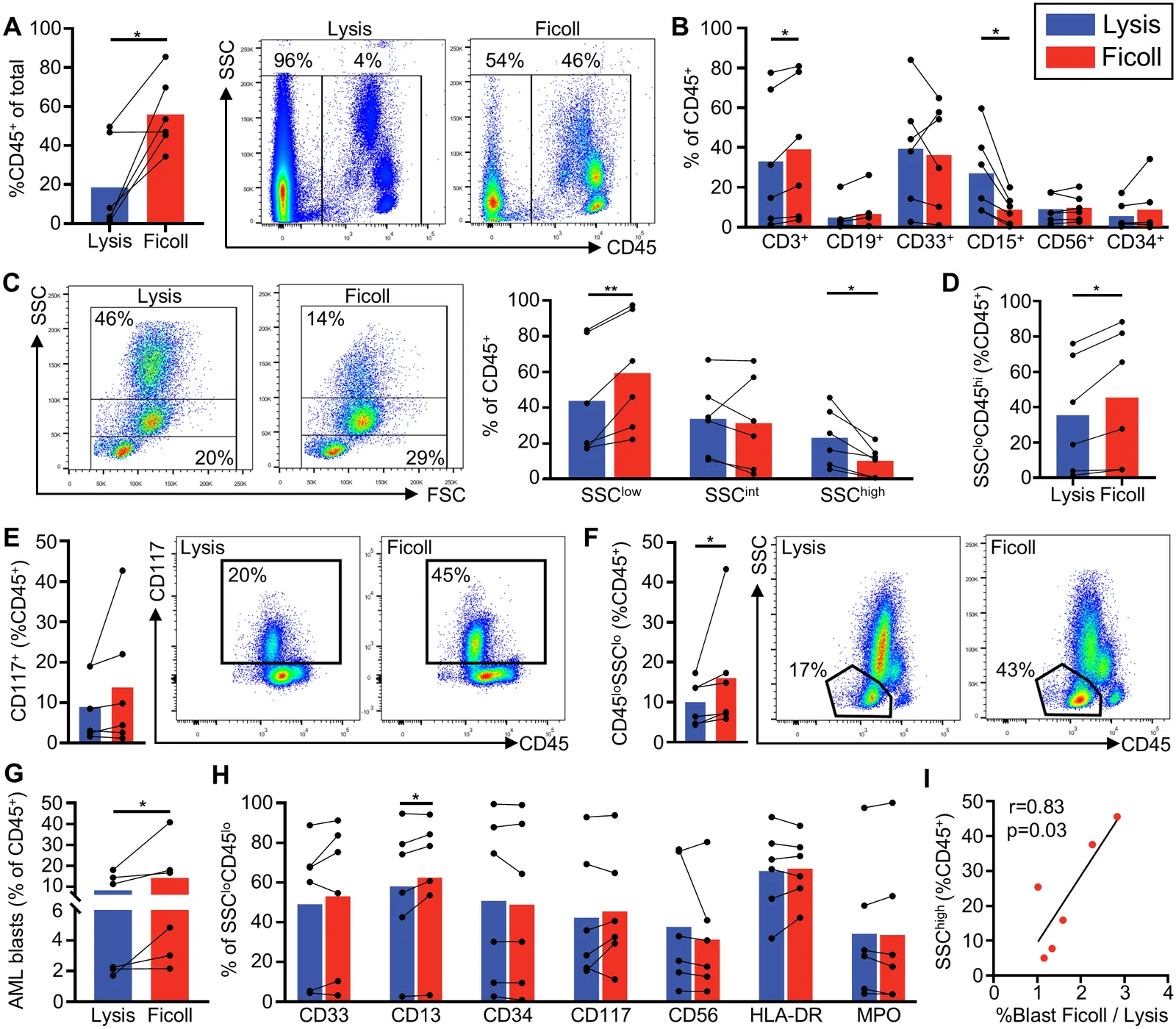
Enrichment of AML blasts and lymphocytes by Ficoll isolation. Leukocytes were isolated from the peripheral blood of six AML patients by either hemolysis or Ficoll-based gradient centrifugation and analyzed by flow cytometry. Paired t-tests were used for comparisons, where each line represents a patient in the bar plots. **(A)** Frequency of leukocytes (CD45^+^ cells) among all detected events. **(B)** Frequency of the indicated markers among leukocytes. **(C)** Flow cytometry dot plot comparing the frequency of leukocytes segregated according to their granularity (side-scatter, SSC). **(D)** Frequency of lymphocytes (gated as SSC^low^CD45^high^ cells) among leukocytes. **(E)** Flow cytometry dot plot and comparison of the frequency of CD117^+^ cells among leukocytes. **(F)** Flow cytometry dot plot and comparison of the frequency of AML blasts (gated as SSC^low^CD45^low^ cells) among leukocytes. **(G)** Frequency of AML blasts among CD45^+^ cells, computed through a Boolean clinical-grade gating strategy. **(H)** Frequency of the indicated markers among AML blasts (gated as SSC^low^CD45^low^ cells). **(I)** Pearson correlation between the frequency of cells with high granularity [SSC^high^, from **(C)**] in the Lysis condition and the enrichment of AML blasts (clinical frequencies from panel **(G)**; %blasts_Ficoll_/%blasts_Lysis_) by the Ficoll approach. All bar plots show the mean (**p<0.01, *p<0.05; paired t-test).

We first analyzed the effects of Ficoll isolation on leukocyte purity (CD45^+^ cells) and noticed that Ficoll samples were significantly enriched with leukocytes, while Lysis samples consisted of substantially more cell debris (**Figure 1A**). After gating on viable cells and excluding debris and doublets, we then analyzed the frequency of various markers within the leukocyte compartment (encompassing lymphocytes, monocytes, granulocytes, and AML blasts) and observed that Ficoll significantly increased the proportion of CD3^+^ T cells while depleting CD15^+^ neutrophils (**Figure 1B**). This imbalance resulted from the depletion of SSC^high^ cells (mainly neutrophils) and the selection of SSC^low^ cells (mainly lymphocytes and blasts) by the Ficoll method (**Figure 1C**). Consequently, the frequency of lymphocytes (SSC^low^CD45^high^) significantly increased after Ficoll isolation (**Figure 1D**). No significant impact was found on the frequencies of CD19^+^, CD56^+^, CD33^+^, and CD34^+^ cells (**Figure 1B**).

In addition, Ficoll increased the frequency of CD34^+^ cells in four patients (**Figure 1B**). The analysis of CD117^+^ cell frequencies revealed a similar trend, with increases observed in the same four patients (**Figure 1E**). Because CD34 and CD117 are the most commonly used markers of AML blasts, we examined these cells more specifically. SSC^low^CD45^low^ cells, an immature phenotype widely shared by AML blasts (39), presented a significantly increased frequency after Ficoll isolation (**Figure 1F**). A clinical-grade analysis of the blasts based on a Boolean gating strategy using CD34, CD117, HLA-DR, CD13, CD7, CD56, and CD45 expression [ELN staining (1)] confirmed the enrichment of AML blasts by the Ficoll method (**Figure 1G**). Given that blasts are SSC^low^ cells, this enrichment probably also results from the relative depletion of SSC^high^ cells by Ficoll. Importantly, no striking impact on the phenotype of blasts was highlighted by these analyses (except for a modest but significant effect on CD13), suggesting that the composition of the blast population is largely preserved in patients with conventional (SSC^low^) blasts (**Figure 1H**). However, substantial patient-specific changes in certain markers were observed, suggesting that Ficoll may alter blast phenotypes unpredictably in some cases. Interestingly, a significant correlation between the frequency of SSC^high^ cells in the Lysis samples and the degree of blast enrichment by the Ficoll method was observed (**Figure 1I**), suggesting that the Ficoll isolation method impacts the composition of the AML samples with abundant granulocytic cells.

Altogether, these results show that the Ficoll isolation method significantly distorts the composition of AML samples through the depletion of granulocytes and the enrichment of lymphocytes and leukemic blasts.

### Ficoll biases the transcriptional and immunogenomic profiling of AML samples compared with hemolysis

Over the last decade, multiple large-scale transcriptomic studies were conducted in AML by consortia such as TCGA, Leucegene, or BEAT-AML. Typically, such studies involve performing RNA-seq on leukocyte fractions. Frequently, the method to isolate these fractions is not specified, and the comparison of results from one cohort to another could be biased by the usage of divergent isolation methods. Additionally, the use of Ficoll may lead to biased estimations of the expression of biomarker candidates or of the abundance of specific immune cell populations in immunogenomic analyses. We therefore investigated whether Ficoll isolation impacts the transcriptional profiling of AML samples in bulk RNA-seq analyses in comparison with the hemolysis approach.

**Figure 2:**
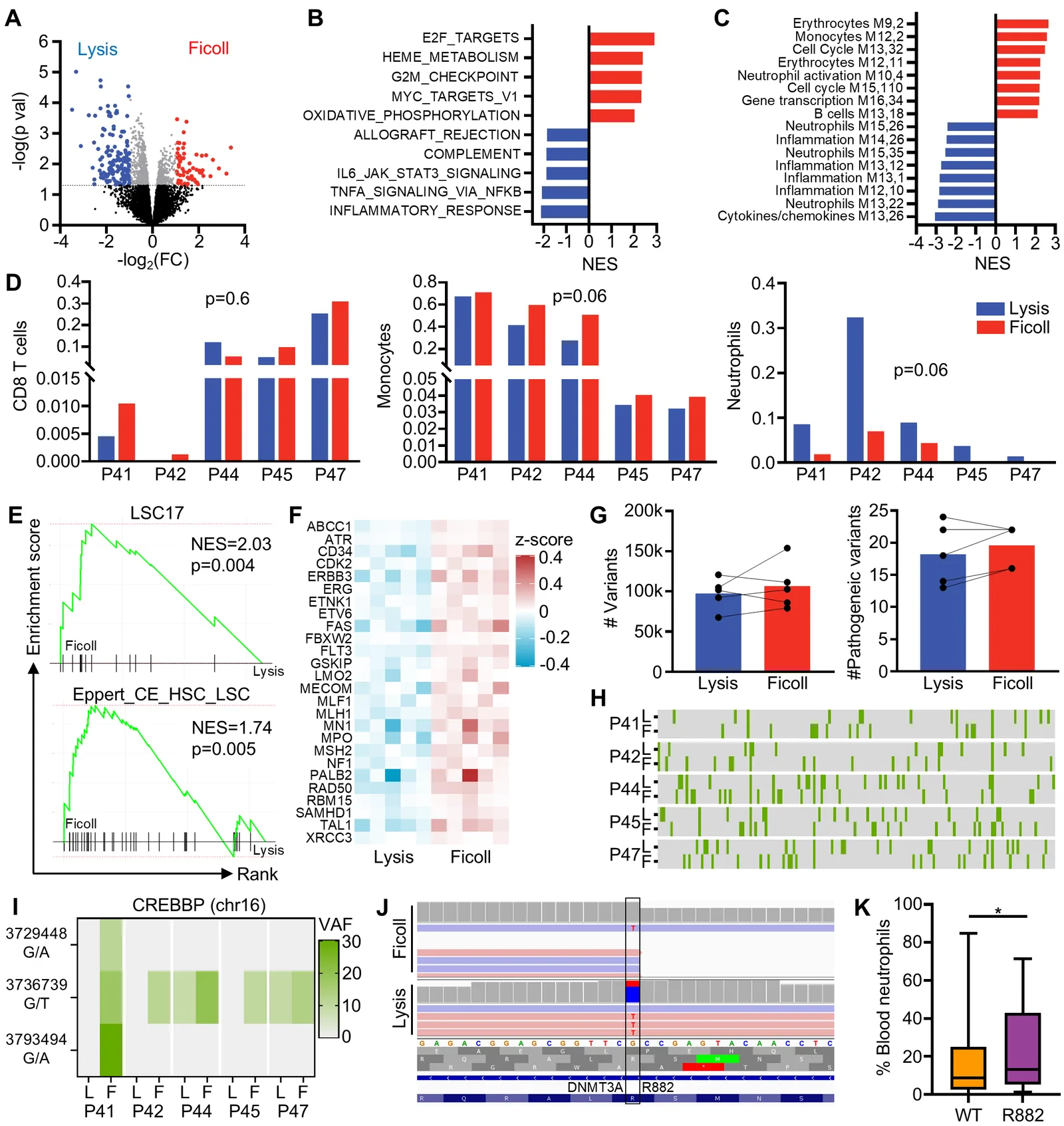
Transcriptional impact of the Ficoll isolation method. Leukocytes were isolated from the peripheral blood of five AML patients by hemolysis or Ficoll-based gradient centrifugation. RNA-seq was performed on total leukocyte fractions. **(A)** Volcano plot of the paired limma-voom analysis performed on bulk RNA-seq data. Significant (p<0.05) genes are colored in red (log_2_(FC)>1), blue (log_2_(FC)<1) or grey. **(B, C)** Paired GSEA with the HALLMARK **(B)** or BTM **(C)** matrices. Gene sets upregulated in Ficoll samples are indicated in red and those upregulated in Lysis samples are indicated in blue. All gene sets are significantly (FDR<0.05) differentially regulated. **(D)** CIBERSORTx deconvolution analysis of the abundance of CD8^+^ T cells, monocytes, and neutrophils in RNA-seq data. **(E)** Paired GSEA of indicated gene sets. **(F)** Heatmap of z-scores of indicated genes in the Lysis and Ficoll samples. **(G)** Total (left) and pathogenic (right) variant counts in Ficoll and Lysis samples. **(H)** Heatmap of the distribution of pathogenic variants (each column is a different variant) in the five patients tested. L, Lysis; F, Ficoll. **(I)** Heatmap of the variant allele frequency (VAF) of the three mutations in the CREBBP gene in the five patients tested. L, Lysis; F, Ficoll. **(J)** IGV visualization of the R882 mutation in the DNMT3A gene of P44. **(K)** Flow cytometry analysis of neutrophils in the blood of DNMT3A R882-mutated patients in the BEAT-AML cohort (*p<0.05, Mann-Whitney test).

RNA was extracted directly from the total leukocyte fraction isolated after the last wash from either the Lysis or Ficoll procedures, consistent with the approach used in the BEAT-AML program (14). Samples from five patients had RNA quality scores sufficient to perform sequencing. Paired differential gene expression analysis showed that 1,136 genes were differentially regulated (p<0.05), with 152 and 59 being highly (FC > 2 or < -2) overexpressed and underexpressed, respectively, in Ficoll samples (**Figure 2A**; **Supplementary Table 1**). This revealed that Ficoll isolation alters the transcriptomic profile of AML samples in comparison with hemolysis isolation.

We then performed GSEA with HALLMARK (40) and BTM (34) gene sets. HALLMARK analysis highlighted lower normalized enrichment scores of inflammatory pathways while proliferation-related pathways were enriched in Ficoll samples, likely reflecting the enrichment of AML blasts in these samples (**Figure 2B**). BTM analysis suggested a depletion of neutrophils and inflammation pathways in Ficoll samples, as well as an enrichment of erythrocytes, monocytes, and B cells (**Figure 2C**). The underexpression of HALLMARK inflammation pathways probably reflected the lower abundance of neutrophils in Ficoll samples, as suggested by BTMs. The enrichment of B cells, monocytes, and erythrocytes was in agreement with our flow cytometry results and the absence of a hemolysis step before RNA extraction in the Ficoll procedure.

Because the two isolation methods yielded markedly different cellular compositions, we questioned whether these differences would be detectable in our RNA-seq data, as suggested by the BTM analysis. Immunogenomic deconvolutions of the cell type composition with CIBERSORTx (41) indicated a lower abundance of neutrophils and a trend toward enrichment of monocytes and CD8^+^ T cells by the Ficoll (**Figure 2D**). Assuming that the enrichment of monocytes may result from the greater abundance of AML blasts, we performed a GSEA analysis with two gene sets specific to LSCs (42, 43) and observed an upregulation of both gene sets (**Figure 2E**). Accordingly, the expression of many AML-related genes (such as CD34, FLT3, and MPO) was upregulated in Ficoll samples (**Figure 2F**). Altogether, these results show that, compared with hemolysis, Ficoll isolation leads to an artificial increase in the abundance of leukemic biomarkers since granulocyte depletion removes granulocyte-derived transcripts that overshadow the transcriptome of hemolysis samples. Ficoll also alters the estimated abundance of multiple immune cell populations compared with hemolysis.

### Ficoll isolation impacts the detection of mutations in RNA-seq samples

Analyses of mutation expression in cancer patient cohorts is frequently performed on bulk RNA-seq data. The expression of such mutations can then be related to patient survival (44), neoantigen generation (45), or the oncogenic process itself (46). We therefore investigated whether the Ficoll method could have an impact on such analyses. After variant calling, a similar number of variants were detected between both Ficoll and hemolysis (**Figure 2G**). An average of 32% of variants were commonly detected, and neither method enriched the number of specific variants. To discriminate the variants most likely to be relevant to AML biology, we annotated them with the ClinVar database (47), and focused on pathogenic and likely pathogenic variants (**Supplementary Table 2**; **Figure 2G**). In agreement with an enrichment of blasts by the Ficoll approach, a trend toward more pathogenic variants was found in Ficoll samples. The identity of detected mutations changed dramatically between approaches, with an average of only ~11% common between both approaches (**Figure 2H**), highlighting the high impact that the isolation method can have on such analyses.

Next, we evaluated whether we could identify genes for which results would differ dramatically between methods. Surprisingly, pathogenic mutations in the CREB-binding protein (CREBBP, also known as CBP) gene were abundantly detected in our dataset. CREBBP is a transcriptional coactivator frequently mutated in hematological malignancies (48, 49), and inactivating mutations in CREBBP can promote leukemogenesis and predict poor prognosis in AML (50, 59). While these mutations were detected in all patients, only Ficoll enabled their detection in three patients, and higher variant allele frequencies (VAFs) were observed in Ficoll samples for the other patients (**Figure 2I**). Because Ficoll increases the proportion of AML blasts, the improved detection of CREBBP mutations possibly reflects the higher fraction of RNA-seq reads originating from leukemic blasts.

Another striking observation was the R882 mutation in the DNMT3A gene, which was detected specifically in the Lysis sample of P44 (**Figure 2J**), in agreement with the reported mutational profile of this patient (**Table 1**). Manual examination revealed a VAF of 27% with a coverage of 37 reads in the Lysis sample, while VAF was 8% with a coverage of 13 reads in the Ficoll sample. The low VAF and coverage in the Ficoll sample probably prevented the adequate calling of the variant. Interestingly, a recent report showed that R882 mutations promoted a greater differentiation of hematopoietic stem cells into neutrophils (51). We therefore hypothesize that the R882 mutation detected herein was expressed in neutrophils (in addition to blasts), which enabled a better detection of the mutation in the Lysis sample (this patient presented low blast percentages in both Lysis and Ficoll samples, with 1.71 and 4.85%, respectively). Accordingly, patients with R882 mutations in the BEAT-AML cohort also showed higher levels of circulating neutrophils, as measured by flow cytometry (**Figure 2K**).

Altogether, these results show how sample composition alterations by Ficoll isolation can bias the detection of specific mutations relevant to AML biology and diagnosis.

### Limited impact on immunogenomic ranking analyses

The availability of large RNA-seq datasets has spurred immunogenomic studies of immune cell abundance, particularly T cells, in the blood and bone marrow of AML patients (18, 20, 52). These analyses are typically performed using deconvolution tools such as CIBERSORTx and often compare the estimated abundance of immune cells between different samples. While our results in **Figures 2C, D** show that Ficoll leads to a misestimation of the intra-sample abundance of some immune populations in RNA-seq samples relative to their real abundance within the leukocytic compartment (assessed with hemolysis), we wondered whether Ficoll isolation could also affect inter-sample comparisons of immune cell abundance.

**Figure 3:**
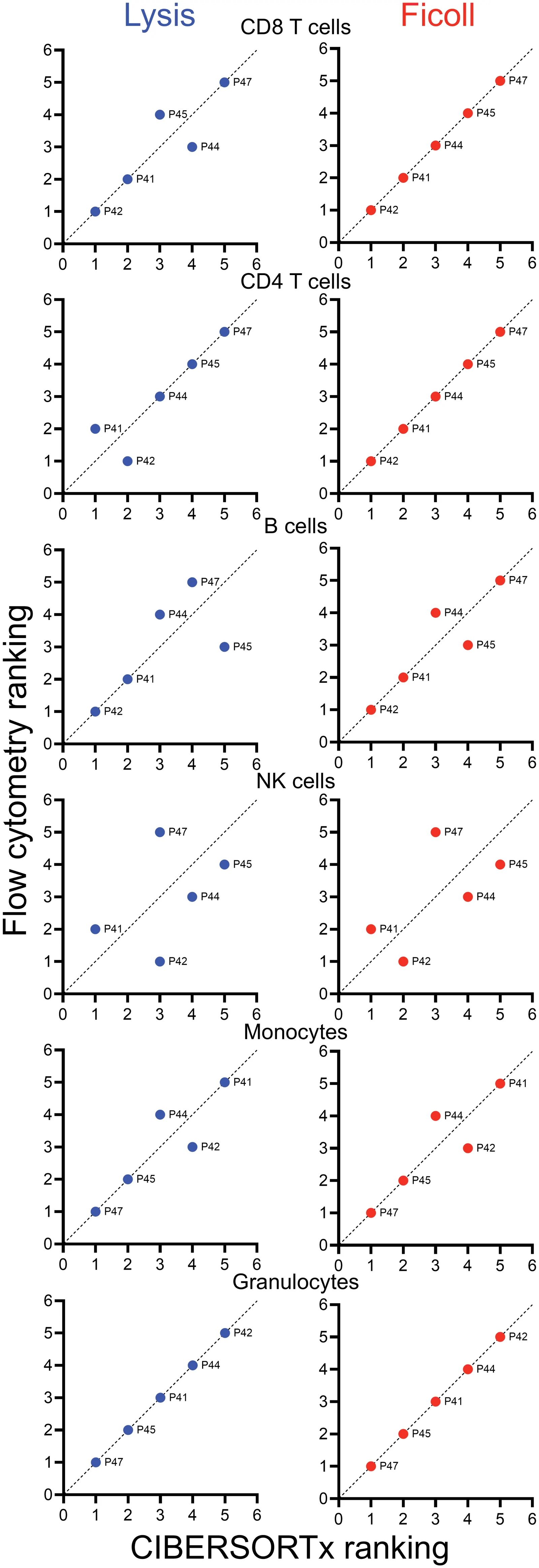
No impact of the Ficoll method on inter-sample immunogenomic comparisons. CIBERSORTx deconvolutions performed on RNA-seq data of leukocytes isolated with each method were used to rank samples from patients, and this ranking was compared to the ranking obtained from flow cytometry analyses performed on Lysis samples (the least biased method for measuring the frequency of each cell population). Samples deviating from the identity diagonal line have discordant rankings between RNA-seq and flow cytometry analyses.

To assess this, we ranked the five sequenced Ficoll samples according to the abundance of each immune cell population deconvoluted with CIBERSORTx and compared these rankings with those obtained based on flow cytometry measurements in Lysis samples. The same was done for the five Lysis samples. As expected, the rankings showed good concordance for CD8^+^ and CD4^+^ T cells, B cells, monocytes, and granulocytes in the Lysis samples (**Figure 3**). A poor concordance was found for NK cells, possibly because of a lower capacity of CIBERSORTx to deconvolute NK cells compared to other cell populations. Fortunately, a good concordance was also found for Ficoll samples, showing that although Ficoll introduces a misestimation of the abundance of some immune cells relative to hemolysis, it does not seem to overly affect inter-sample comparisons. This suggests that previous immunogenomic studies conducted on Ficoll-isolated samples are probably not overly biased by the use of this isolation procedure.

### T-cell enrichment by Ficoll isolation might affect *in vivo* AML blast expansion

**Figure 4:**
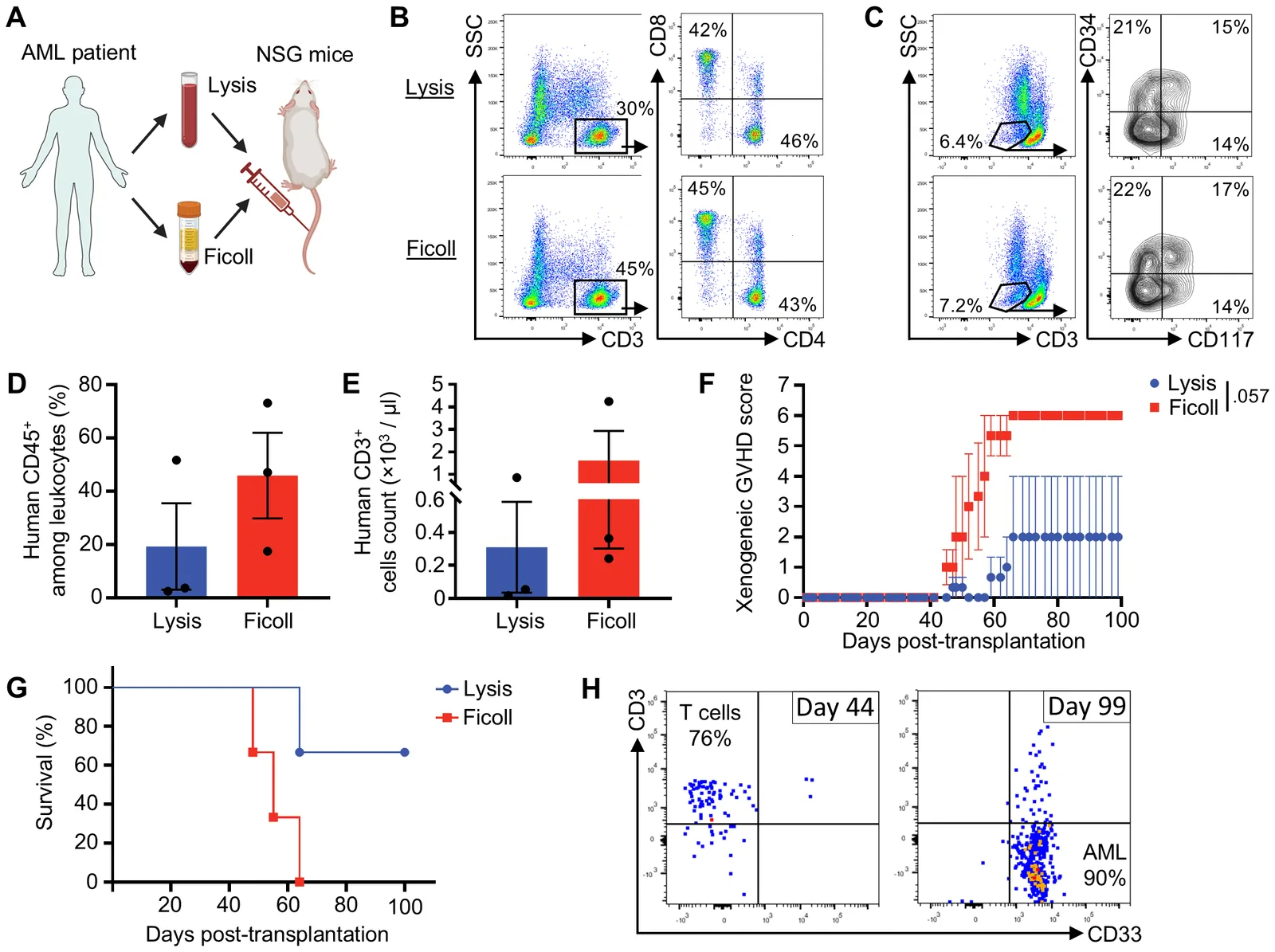
Impact of the Ficoll isolation method on AML blast expansion *in vivo*. Leukocytes were isolated from the peripheral blood of one AML patient (P46) by either hemolysis or Ficoll-based gradient centrifugation. The total leukocyte fraction was injected into NSG mice 24 h after non-lethal irradiation. **(A)** Experimental design. **(B)** Flow cytometry analysis of the frequency of T cells (CD3^+^) and their subsets, CD4^+^ and CD8^+^ cells, among the leukocytes before injection into NSG mice. **(C)** Flow cytometry analysis of the frequency of AML blasts (SSC^low^CD45^low^) among leukocytes before injection into NSG mice, with CD34 and CD117 expression. **(D)** Frequency of human CD45^+^ cells among leukocytes (_human_CD45 + _mouse_CD45 cells) in the peripheral blood of NSG mice on day 42. **(E)** Concentration of human CD3^+^ cells in the blood of NSG mice on day 42. **(F)** Scoring curve of GVHD symptoms in NSG mice over the entirety of the experiment (ANOVA-2). **(G)** Log-rank survival curve; mice were sacrificed upon reaching an ethical score limit of 6. **(H)** Flow cytometry analysis of the frequency of T cells and myeloid cells in the blood of NSG mice on days 44 and 99.

Next, we tested whether the alterations of cell population abundances by Ficoll may impact the functional characterization of AML samples. AML blast enrichment before transplantation is frequently performed to establish patient-derived AML xenografts in immunodeficient mice (15, 25, 53, 54). However, the infusion of unfractionated Ficoll-isolated mononucleated cells is also performed (18, 55–59). Given the impact of the Ficoll isolation method on AML and non-AML cell frequencies, we reasoned that it may alter AML engraftment efficacy in immunodeficient mice. Using the cells of P46 for which we had enough material post-isolation, we transplanted one million leukocytes after Ficoll or Lysis isolation to three mice per condition (**Figure 4A**). In this patient, the CD45^+^ cell population presented similar frequencies of AML blasts (6.4% vs. 7.2% in Lysis and Ficoll, respectively) while Ficoll enriched for T cells by 15% (**Figures 4B, C**). Therefore, mice transplanted with AML cells isolated through Ficoll also received proportionally more T cells (4.5 ×10^5^) than Lysis-engrafted ones (3 ×10^5^). These T cell quantities are typically considered insufficient to induce xenogeneic GVHD upon injection into NSG mice (60).

Flow cytometry analyses showed that Ficoll mice presented a trend toward greater engraftment of human CD45^+^ cells and greater abundance of CD3^+^ T cells (**Figures 4D, E**). This higher T-cell abundance was likely responsible for the development of unexpected GVHD symptoms that we observed in the three mice transplanted with these cells (**Figure 4F**). These mice were euthanized between days 48–64 upon reaching the typical ethical limit score of GVHD symptoms (**Figure 4G**). In contrast, only one mouse transplanted with a Lysis sample required sacrifice on day 64 (due to GVHD), and the two other mice survived until the end of the experiment (day 100). To support the implication of GVHD in the death of these four mice, we analyzed the frequency of T cells in their blood, either on a day close to sacrifice for two of them (four and nine days before sacrifice, respectively) or at necropsy for the remaining two. This showed that CD3^+^ T cells represented the main human cell population (73-97% of CD45^+^), while CD33^+^ AML cells represented less than 1% (**Supplementary Figure 2**). The same observation was made in the bone marrow and spleen of necropsied animals (**Supplementary Figure 3**). Furthermore, analysis of the CD3^+^ compartment in the spleen and bone marrow revealed the presence of CD4^+^CD8^+^ double-positive T cells, a biomarker of GVHD (61, 62).

In contrast, flow cytometry analysis on the peripheral blood of the two surviving hemolysis mice on day 100 showed that one of them presented a high frequency (90%) of CD3^─^CD33^+^ cells, which were most likely AML cells since normal myeloid cells do not survive and expand in NSG mice (60) (**Figure 4H**). This mouse presented only circulating T cells on day 44, suggesting that the failed long-term engraftment of T cells enabled the expansion of AML cells. The other mouse presented a frequency of 75% of CD3^─^CD33^+^ AML cells (**Supplementary Figure 4**).

### Limited impact of Ficoll isolation on *in vitro* AML blast expansion and chemotherapy response

**Figure 5:**
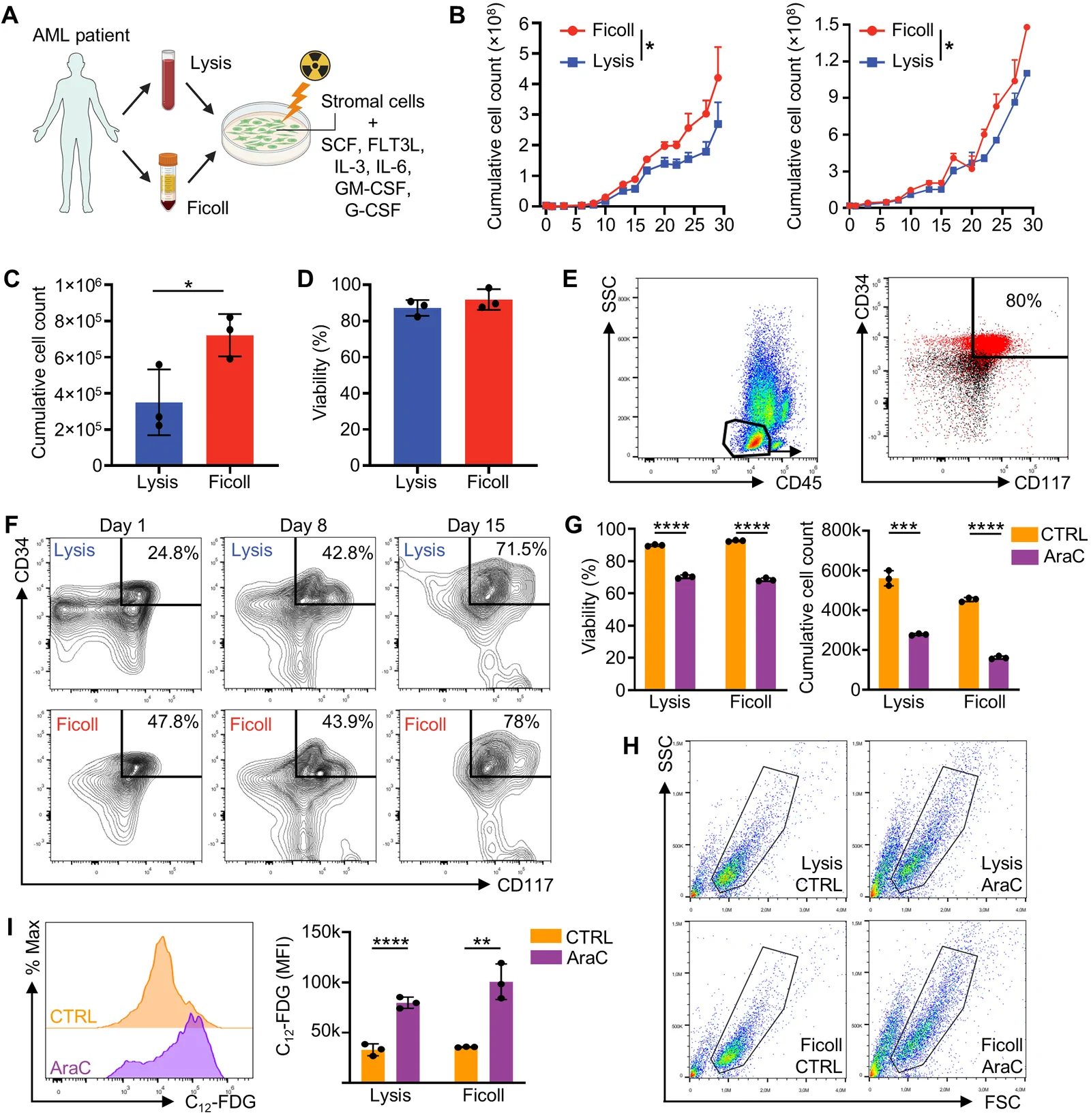
Impact of the Ficoll isolation method on AML blast expansion *ex vivo*. Leukocytes were isolated from the peripheral blood of four AML patients by either hemolysis or Ficoll-based gradient centrifugation. AML blasts were then expanded *ex vivo* for four weeks. **(A)** Experimental overview. **(B)** Cumulative count of viable (7-AAD^─^) cells from patient 42 (left) and 44 (right) over the course of the culture. ANOVA-2 tests were used to compare expansion between the two conditions. **(C)** Cumulative count of viable (7-AAD^─^) AML blasts (SSC^low^CD45^low^) after 72 h of culture. **(D)** Viability (% of 7-AAD^─^) of AML blasts (SSC^low^CD45^low^) after 72 h of culture. **(E)** CD34 and CD117 expression of SSC^low^CD45^low^ blasts (in red) and non-blasts (in black) after 24 h of culture. The percentage represents the frequency of CD34^+^CD117^+^ cells among SSC^low^CD45^low^. **(F)** Evolution of CD34^+^CD117^+^ cells among CD45^+^ cells at indicated time points. **(G)** Viability (% of 7-AAD^─^) and cumulative cell count of cultured AML blasts treated *ex vivo* with 1.5 µM AraC for 72 h. **(H)** Cell size (FSC) and granularity (SSC) of AML blasts after 72 h of AraC treatment. **(I)** Flow cytometry histogram depicting SA-β-Gal activity using the fluorogenic β-galactosidase substrate C_12_-FDG, gated on viable cells (7-AAD^─^), after 72 h of AraC treatment. All bar plots show the mean ± SD (****p<0.0001, ***p<0.001, **p<0.01, *p<0.05; t-test).

Cell lines do not reflect the epigenetic state of primary cancer cells (63) or their mutation spectrum (64). Therefore, primary cell culture of AML blasts is increasingly used, notably to study AML response to chemotherapy (55). In light of our results, we reasoned that Ficoll isolation could alter the efficacy of such cultures either by reducing (due to the presence of more lymphocytes in the isolate) or promoting (due to the presence of more AML blasts in the isolate) the AML expansion rate. Therefore, from the cells of four patients for which enough material was available, we performed *ex vivo* expansion of the total leukocyte population by culturing AML blasts on a layer of irradiated bone marrow stromal cells (OP9) in the presence of a cytokine cocktail (**Figure 5A**). The cells from two patients (P42 and P44) eventually proliferated, in agreement with the success rate (~55%) of this culture system reported previously (17).

Interestingly, Ficoll-isolated AML cells proliferated slightly but significantly better than Lysis-isolated cells from both patients (**Figure 5B**). Since both patients displayed comparable results, we focused on patient 42, where the largest differences were observed (although all subsequent findings were also confirmed in patient 44). Flow cytometry analysis 72 h after the start of the culture showed that Lysis had lower SSC^low^CD45^low^ AML blasts (**Figure 5C**), in agreement with the lower starting percentage of blasts in these samples. No difference in cell death was observed between the two conditions (**Figure 5D**). Therefore, the slower growth in the Lysis condition probably resulted from the lower initial abundance of AML blasts rather than from a greater cell death by the hypotonic shock of the Lysis procedure.

We then questioned whether the isolation method might affect the immature/stem cell phenotype of the blasts over time and observed that the SSC^low^CD45^low^ blasts from this patient were CD34^+^CD117^+^ (**Figure 5E**). We therefore analyzed the frequency of CD34^+^CD117^+^ cells among total leukocytes (CD45^+^ cells) at three time points (days 1, 8, and 15) and observed that their frequency tended to increase with time (especially in the Lysis condition), showing that this population was expanding and that our culture system preserved their leukemic phenotype (**Figure 5F**). On days 8 and 15, no difference in blast frequency was found between Ficoll- and Lysis-isolated blasts. Altogether, these results suggest that the lower frequency of AML blasts in Lysis samples at the start of the culture is responsible for the mild delay of expansion, but this does not eventually impact the AML cell phenotype in the culture.

Next, we assessed whether blasts expanded from both isolation methods respond similarly to chemotherapy *in vitro*. AraC was tested since it is the main drug given as first-line chemotherapy to patients in the conventional 7 + 3 regimen. Blasts were collected from our primary cultures on day 21 of expansion and were treated with AraC. At a dose of 1.5 µM, AraC significantly reduced the cell counts and viability of AML cells (**Figure 5G**). In agreement with a previous report assessing AraC effects on primary AML cultures (55), AraC increased the cell size and granularity (**Figure 5H**). Another key feature of the relapse-mediating AML post-chemotherapy is the expression of Senescence-Associated(SA)-β-Galactosidase (55). We therefore stained our cells with the fluorogenic substrate of the SA-β-Gal, C_12_-FDG (65), and observed that AraC increased its activity, as expected (**Figure 5I**). Importantly, no significant difference in AraC effect on SA-β-Gal was observed between isolation methods, in agreement with the comparable blast phenotype between conditions.

### Limited impact of leukocyte isolation method on cryopreservation

In laboratory practice and biobanking, leukocyte isolation is frequently followed by long-term cryopreservation. We therefore evaluated the effects of cryopreservation on samples processed by either Ficoll or Lysis. This comparison was performed with three patient samples for which sufficent cells remained after completing the analyses described in the previous sections. These cells were cryopreserved in FBS containing 5% DMSO and kept in liquid nitrogen for 16–18 months. Upon thawing, flow cytometry was conducted to evaluate the abundance of the main cell populations. As shown in **Supplementary Figures 5**, **6**, the frequency of nearly all cell populations was affected by cryopreservation. However, we did not observe any major differences in the effects of cryopreservation between Ficoll- and Lysis-processed samples, suggesting that cryopreservation does not substantially alter the impact of the initial sample processing method under our experimental conditions.

## Discussion

To the best of our knowledge, this study is the first to evaluate the extent of the bias introduced by Ficoll isolation in the profiling of AML samples for research purposes. We demonstrate that Ficoll-based density gradient centrifugation artificially overestimates the abundance of leukemic blasts and lymphocytes while underestimating the abundance of SSC^high^ granulocytes in AML samples. The extent of this distortion varied substantially depending on the analytical method used to profile the AML samples. We therefore advocate the use of hemolysis for studies in which the global cellular composition of AML samples is examined, while Ficoll could be desirable for studies aiming to enrich SSC^low^ leukemic blasts or improve the detection of molecules present in this population. Additionally, our results enable us to make specific recommendations for each type of analysis to be performed on AML samples, depending on the purpose of the investigation. These recommendations are discussed in the following sections.

Flow cytometry is the most broadly used method to characterize the phenotype of AML blasts in the clinic as well as to monitor MRD. While hemolysis is the standard method recommended by the ELN to process AML samples, Ficoll isolation is still broadly used, especially in research. Here, we observed that Ficoll systematically increased the abundance of SSC^low^ AML blasts and lymphocytes making the evaluation of the abundance of the cells largely biased. The impact of Ficoll isolation on AML characterization has previously only been explored with respect to blast phenotype (66), where no differences were detected. However, only binary marker positivity was assessed and not quantitative shifts in population proportions, as performed herein. Importantly, certain AML subtypes, such as KMT2A-rearranged AML and acute promyelocytic leukemia, typically display high SSC values (4), suggesting that their blast composition (unfortunately not represented in our dataset) may be susceptible to Ficoll-induced distortion. Moreover, chemotherapy has been shown to induce eosinophil-like SSC^high^ AML-derived cells capable of mediating relapse (67). Therefore, the use of the hemolysis approach is highly recommended for flow cytometry analyses. In our hands, the main limitation to this approach was the abundance of debris. Since debris can be gated out post-acquisition as CD45-negative cells, it does not pose a major issue in flow cytometry analyses.

Consistent with the flow cytometry results, transcriptomic analyses were heavily biased by Ficoll isolation, with over one thousand genes found to be differentially expressed, mostly reflecting the altered cell proportions observed by flow cytometry. Therefore, Ficoll isolation could lead to inaccurate conclusions about the relative abundance of granulocytes compared with smaller cell populations (AML blasts and lymphocytes) in immunogenomics analyses. Additionally, the artificial enrichment of transcripts derived from AML-related genes could bias biomarker discovery studies. We also observed that Ficoll can alter the detection of AML-relevant mutations. For example, the DNMT3A^R882^ mutation was missed in one sample processed with Ficoll, whereas CREBBP mutations were better detected with Ficoll, which may reflect their preferential expression in AML blasts that are enriched by Ficoll isolation. We hypothesize that the non-detection of the DNMT3A^R882^ mutation resulted from the depletion of neutrophils during Ficoll isolation. Indeed, previous reports showed that neutrophils in AML are dysfunctional and immunosuppressive, thereby increasing the risks of infection and mitigating anti-AML immunity (68). Moreover, AML blast differentiation is not completely blocked, allowing AML-specific mutations to be detected in more mature populations, such as monocytes and neutrophils (69, 70). Thus, the removal of granulocytes by Ficoll isolation represents a significant loss of information for the RNA-seq-based analysis of AML biology. Therefore, despite the absence of major effects on inter-sample immunogenomic rankings in our study, the relative enrichment of lymphocytes and AML blasts together with the depletion of granulocytes introduces an undeniable bias into transcriptomic analyses of AML samples. Consequently, the Lysis method appears preferable for transcriptomic studies, particularly when generating large datasets intended for multiple applications (e.g., immunogenomic analyses, mutation profiling, biomarker discovery, and other downstream investigations). Because Lysis-derived samples may contain substantial cellular debris, we recommend sorting viable leukocytes (CD45^+^7-AAD^−^) before RNA extraction to minimize contamination from dying cells. A previous study showed that such sorting has only a minimal impact on gene expression in the analyzed samples (71). Nevertheless, to prevent sorting-induced stress responses that could alter gene expression, cells should be sorted directly into RNA extraction lysis buffer, where RNA is immediately stabilized (72). Finally, as we observed a trend toward lower detection of pathogenic mutations in Lysis-isolated samples, potentially due to reduced coverage of blast-specific variants, we recommend high-depth sequencing (>50 million reads) to ensure comprehensive coverage of leukemic gene expression and mutation profiles in Lysis-prepared samples.

Regarding xenotransplantations of AML cells, we observed that mice transplanted with Ficoll samples developed GVHD symptoms, while Lysis-isolated samples allowed successful AML engraftment in two out of three mice. While we believe that no major differences between isolation methods would have been observed had we used samples containing >80% blasts, our findings highlight the potential risks associated with Ficoll-induced alterations in sample composition. We therefore recommend the systematic use of hemolysis isolation, combined with lymphocyte depletion as previously described (25), in AML xenograft models to prevent GVHD. Notably, such depletion can also accelerate AML engraftment, even when T cell numbers are below the threshold required to induce GVHD (73). Importantly, T cell depletion is preferable to positive selection of AML blasts (e.g., CD34^+^ selection) because leukemia-initiating cells can reside in multiple subpopulations, including CD34^−^ cells (23), and antibody binding to leukemic cells may impair engraftment efficiency in mice (24).

In contrast with other analytical methods, no striking impact of the Ficoll isolation method was observed on *ex vivo* AML cultures. Although Ficoll samples showed a slight proliferation advantage compared to Lysis samples, cellular phenotype and response to chemotherapy were similar. We surmise that this advantage results from the greater abundance of AML blasts in the Ficoll samples. It is also possible that the greater amount of cellular debris present in Lysis samples negatively affects the AML blasts. While this hypothesis cannot be fully excluded, the absence of a viability difference between the two culture conditions suggests that the debris did not induce significant toxicity to the blasts. While it may be tempting to perform a viable-cell sorting step before culture to eliminate debris, this could impose unnecessary stress on the blasts, potentially causing more deleterious than beneficial effects. Finally, the greater size and granularity observed in AML cells surviving chemotherapy (drug-tolerant persisters) suggest that the Ficoll isolation method could affect the composition of this subpopulation. Since drug-tolerant persisters are considered to represent the main population constituting MRD and mediating subsequent relapse, the use of Ficoll in research focused on these cells is not recommended. In summary, we recommend the use of the hemolysis approach to isolate AML cells before *ex vivo* expansion without further fractionation of the samples.

The present study has several limitations that should be considered when interpreting its findings. The first is the limited statistical power resulting from the inclusion of only six AML samples. Given the marked biological heterogeneity of AML, it is possible that different observations would be obtained in an independent cohort. This is particularly true for the transcriptomic analyses, for which only five patients could be included. The same applies to our mouse experiment, which was based on only three animals per condition and cells from a single AML patient. These analyses should therefore be considered exploratory and will require validation in larger cohorts. The second limitation is that red blood cell lysis may also affect the biological properties of AML samples. In the absence of a technically feasible method to analyze completely unprocessed AML blasts, our study should therefore be interpreted as a comparison of two sample processing methods rather than as an assessment of their absolute fidelity to the *in vivo* biological state.

In conclusion, our work highlights that leukocyte isolation is not a trivial preparatory step but a critical methodological variable that shapes the biological and translational insights derived from AML samples. Our findings advocate for the use of hemolysis as the preferred method for leukocyte isolation in AML research and highlight the necessity to disclose the isolation method used in the preparation of large-scale omics datasets. In contrast, Ficoll may be preferrable when the aim is to enrich SSC^low^ AML blasts and lymphocytes or to improve the detection of molecules expressed by these cell populations.

## Data Availability

The datasets presented in this study can be found in online repositories. The names of the repository/repositories and accession number(s) can be found below: https://www.ncbi.nlm.nih.gov/geo/, GSE313042.
