# Supplementary material for: Ficoll-based isolation biases the profiling of immune cells and leukemic blasts in AML samples
Source: Front Immunol. 2026 Aug 25;17:1920697. doi: 10.3389/fimmu.2026.1920697 (PMC13550220; doi:10.3389/fimmu.2026.1920697)
Supplement: Supplementary file 1 [file DataSheet2.pdf]

Ficoll-based isolation biases the profiling of immune cells and leukemic blasts in AML samples

Bianca E Silva[1,*], Alison Daubry[1,*], Charline Faville[1,*], Adrien De Voeght[2], Jacques Foguenne[3], Mégane Jassin[1], Oswin Kwan[1], Leslie Correia Da Cruz[1], Gabriele Carriglio[1], Sébastien Charles[1], Alexandra Veloso[1], Frédéric Baron[1,2], Jo Caers[1,2], André Gothot[3], and Grégory Ehx[1,4,†]

[1]GIGA Institute, Laboratory of Hematology, University of Liège, Liège, Belgium
[2]Department of Hematology, University Hospital Center of Liège, Liège, Belgium
[3]Department of Laboratory Hematology, University Hospital Center of Liège, Liège, Belgium
[4]Walloon Excellence in Life Sciences and Biotechnology (WELBIO) Department, WEL Research Institute, Wavre, Belgium
* Co-first authors

† Corresponding author

Correspondence: g.ehx@uliege.be

SUPPLEMENTAL DATA

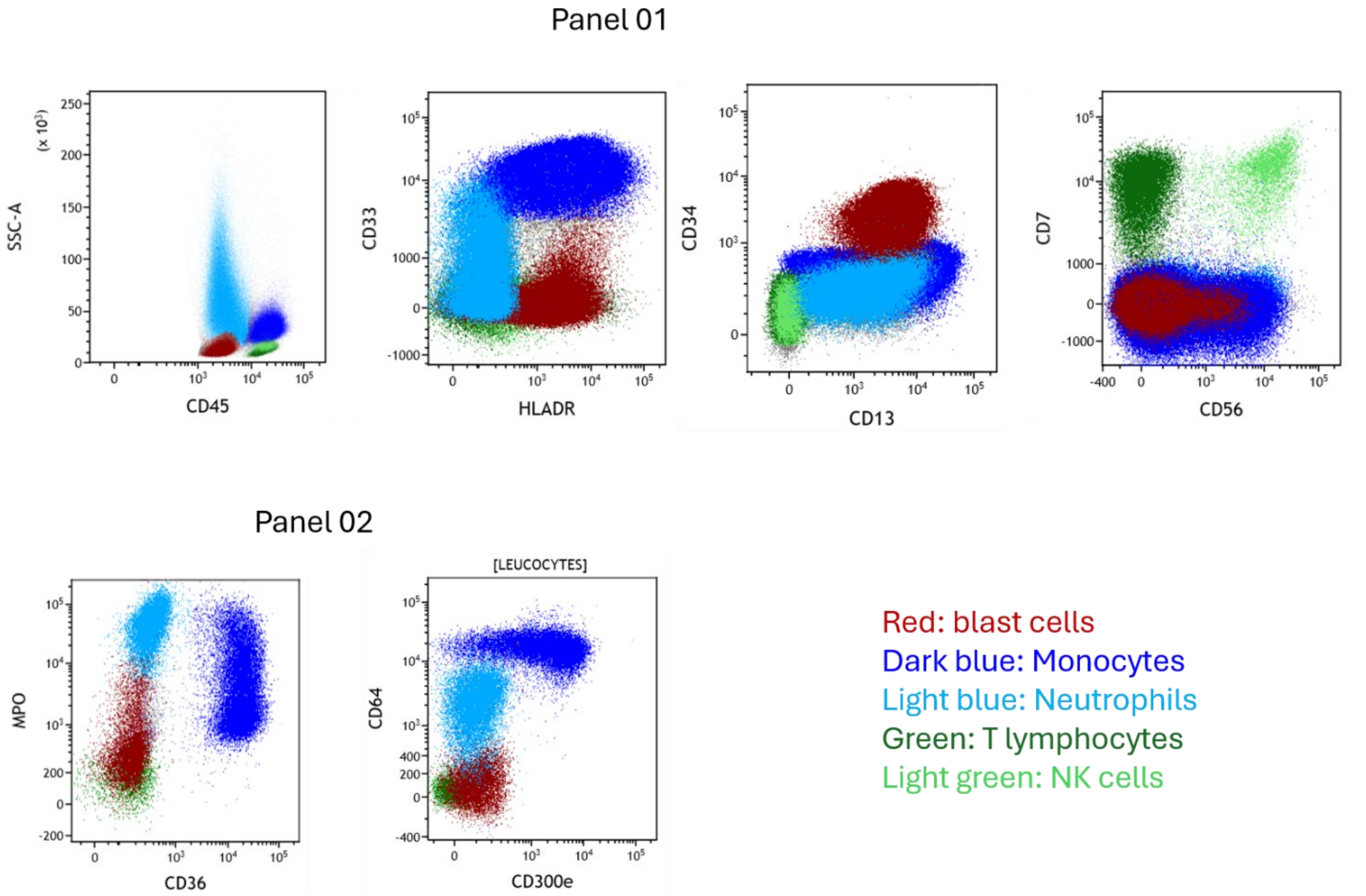

Figure S1. Illustration of the gating strategy used in routine clinical analyses of AML blasts.

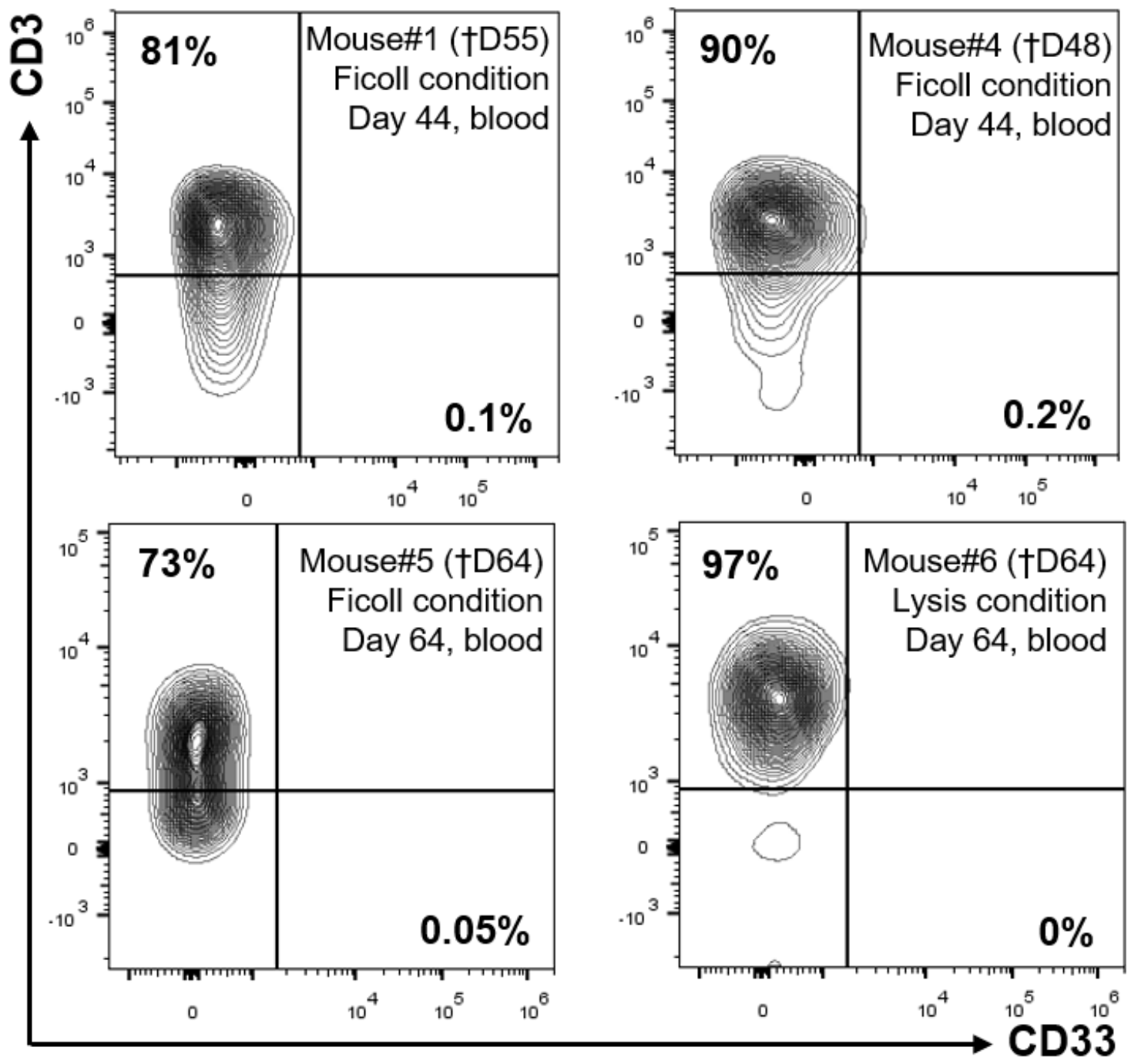

Figure S2. Frequencies of $CD3^+$ and $CD33^+$ cells in the peripheral blood of indicated animals at indicated time points, as analyzed by flow cytometry.

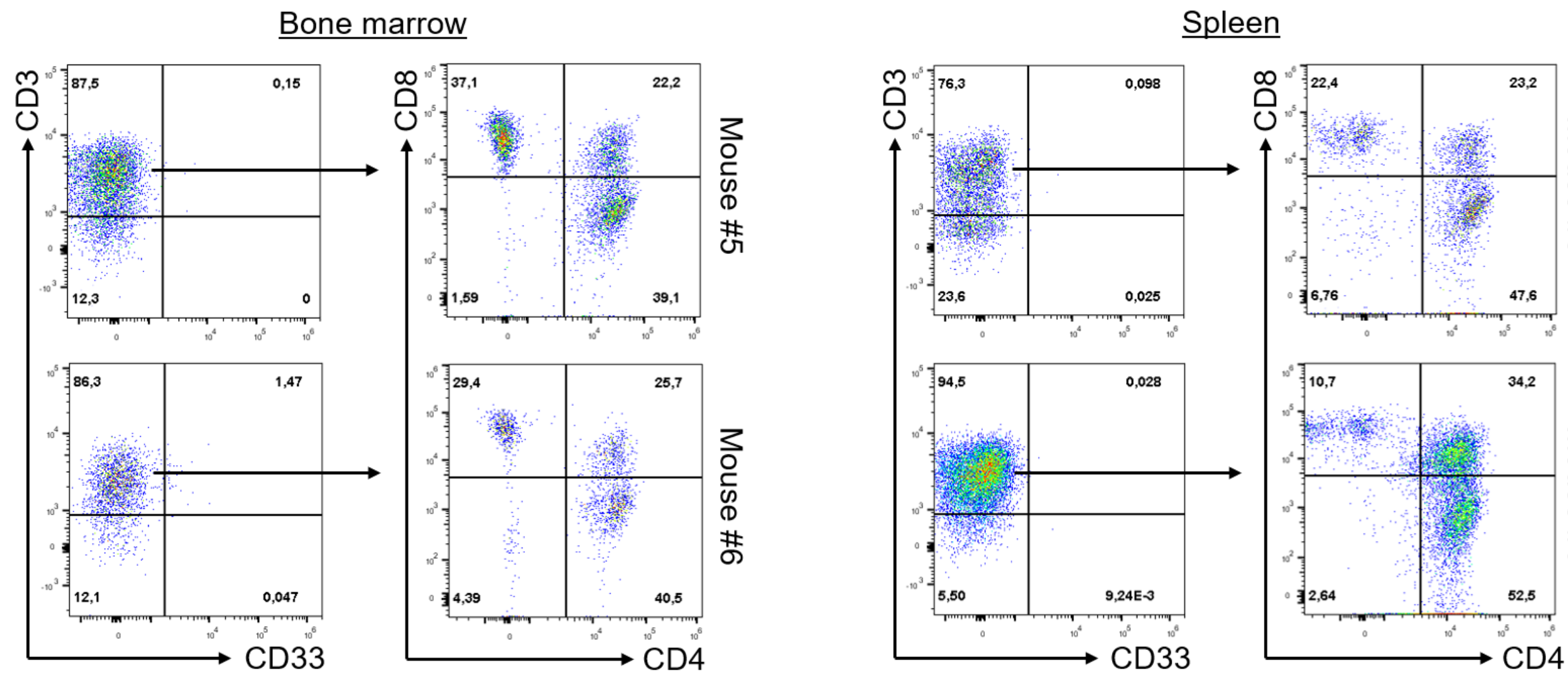

Figure S3. Flow cytometry analysis of $CD3^+$, $CD33^+$, $CD4^+$, and $CD8^+$ cells in the spleen and bone marrow of mice 5 (Ficoll) and 6 (Lysis).

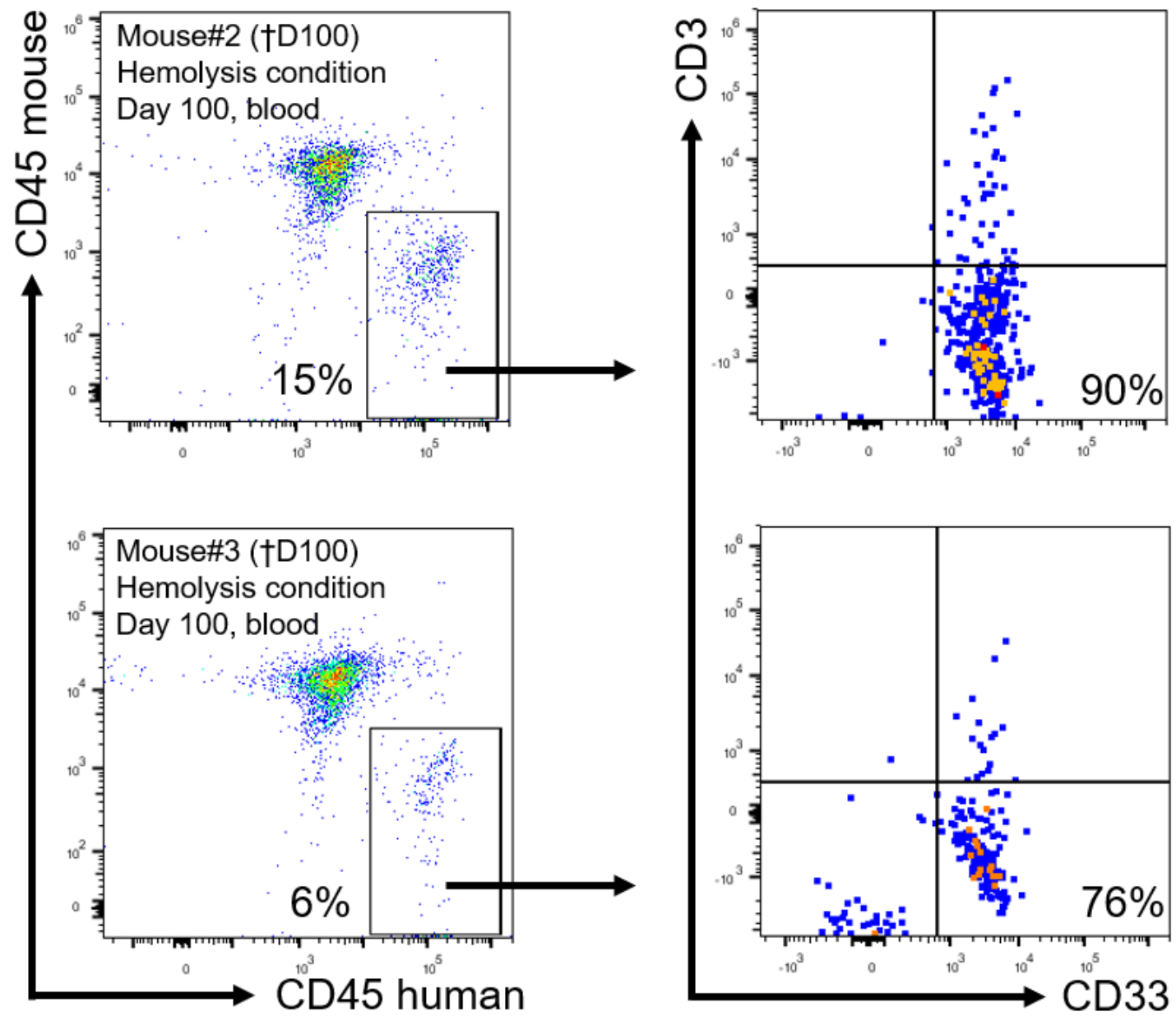

Figure S4. Flow cytometry analysis of mouse and human $CD45^+$ cells and $CD3^+$ and $CD33^+$ cells in the peripheral blood of indicated mice at indicated time points.

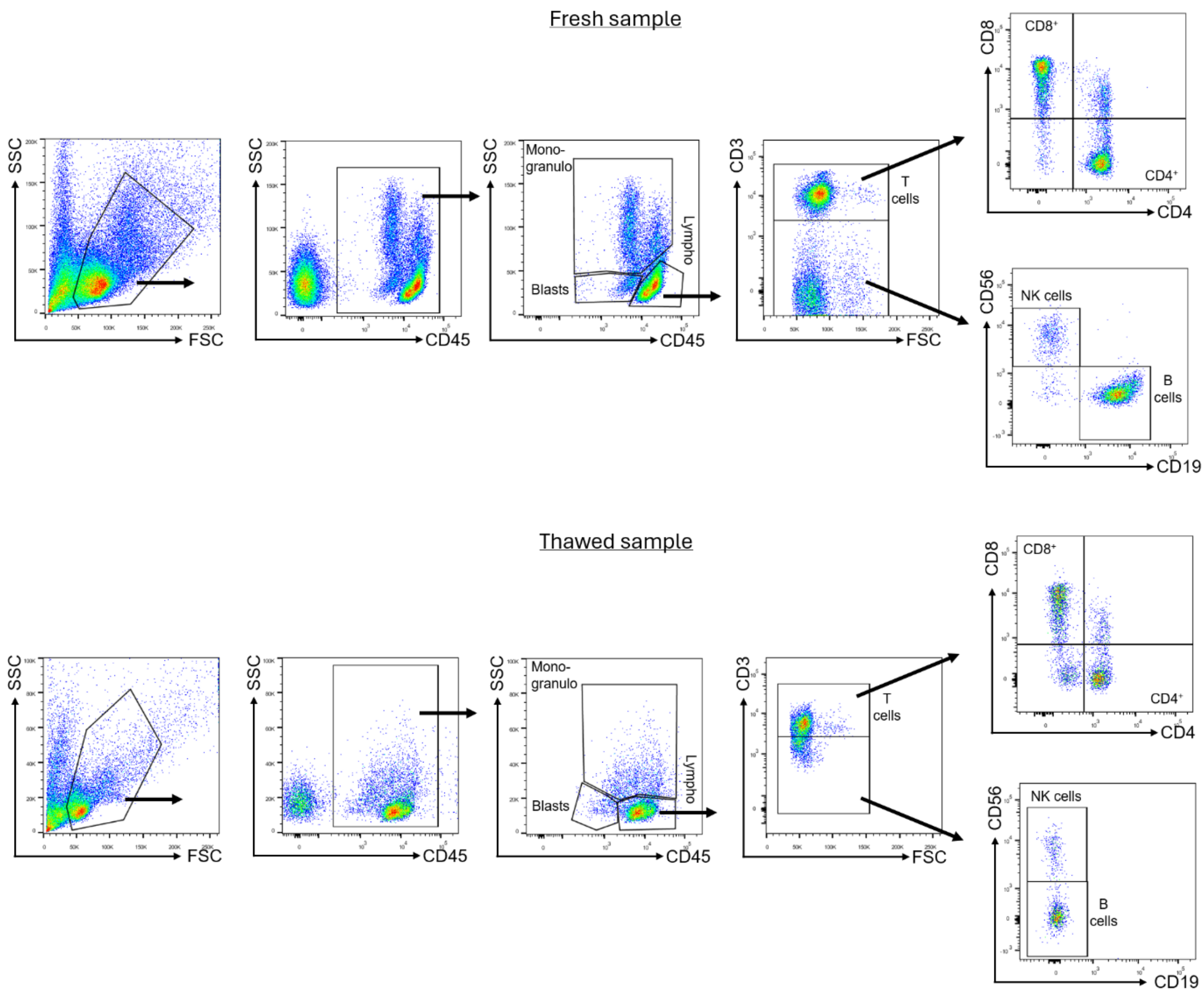

Figure S5. Illustration of the gating strategy used to compare freshly isolated and thawed samples. The sample shown is the Ficoll-isolated specimen from patient 46. This figure illustrates the impact of cryopreservation on the detection and resolution of multiple antigens and cell populations by flow cytometry. The doublet removal step is not shown.

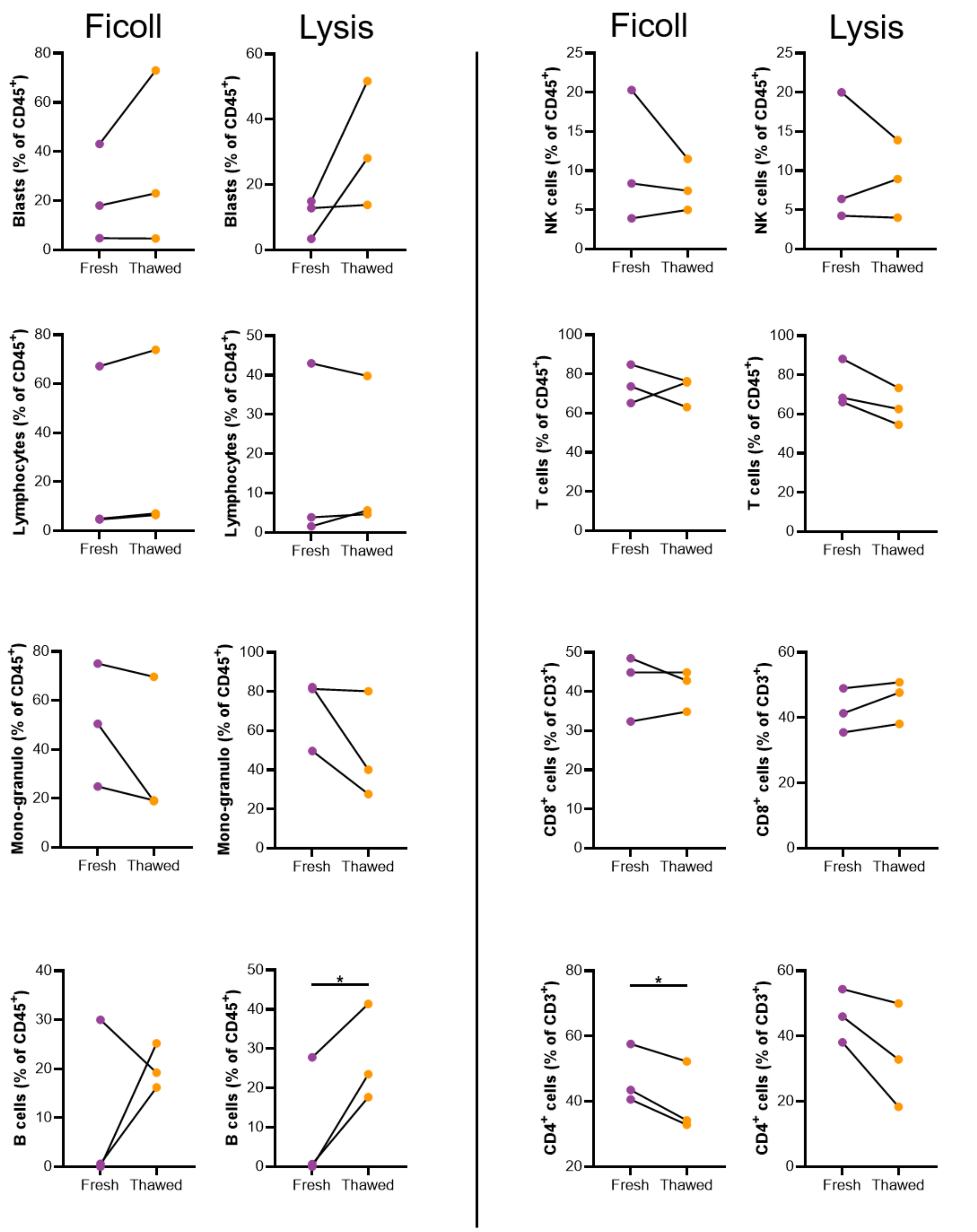

Figure S6. Effects of cryopreservation on the frequency of indicated populations. Leukocyte specimens isolated using Ficoll or hemolysis were cryopreserved in FBS containing DMSO and stored for more than one year before thawing. Flow cytometry was performed according to the gating strategy shown in Figure S5 on freshly isolated (before cryopreservation) or thawed samples (staining immediately after thawing).
